# Editorial: Advances in platforms for disease modelling

**DOI:** 10.3389/fcell.2023.1190492

**Published:** 2023-04-19

**Authors:** Leonardo Romorini, Sukriti Baweja, Rosa Maria Martin Mateos, Gautam Mehta, Nidhi Jalan-Sakrikar

**Affiliations:** ^1^ Laboratorios de Investigación Aplicada en Neurociencias (LIAN-CONICET), Fundación para la Lucha contra las Enfermedades Neurológicas de la Infancia (Fleni), Buenos Aires, Argentina; ^2^ Department of Molecular and Cellular Medicine, Institute of Liver and Biliary Sciences, Delhi, India; ^3^ Servicio de Gastroenterología, Hospital Universitario Ramón y Cajal, Instituto Ramón y Cajal de Investigación Sanitaria (IRYCIS), Universidad de Alcalá, Centro de Investigación Biomédica en Red de Enfermedades Hepáticas y Digestivas (CIBERehd), Madrid, Spain; ^4^ The Roger Williams Institute of Hepatology, Foundation for Liver Research, London, United Kingdom; ^5^ Liver Failure Group, UCL Medical School, Institute for Liver and Digestive Health, University College London, London, United Kingdom; ^6^ Division of Gastroenterology and Hepatology, Center for Cell Signaling in Gastroenterology, Mayo Clinic, Rochester, MN, United States

**Keywords:** big data, sequencing, artificial intelligence, CRISPR, biomarker

In recent years, the development of computational platforms has revolutionized disease modeling and drug discovery, providing researchers with powerful tools to accelerate the identification of new treatments and therapies. Advances in precision medicine and single-cell technologies to understand disease heterogeneity require sophisticated model platforms that can be utilized to gain better insight into disease pathogenesis. These platforms have enabled the integration of large-scale data from diverse sources, such as genomics, proteomics, metabolomics, and spatialomics, allowing for a more comprehensive understanding of disease mechanisms. Moreover, the use of artificial intelligence (AI) and machine learning algorithms has enabled the prediction of drug efficacy and toxicity, reducing the cost and time required for preclinical and clinical trials. This Research Topic aimed to gain insight into various platforms available to study human diseases across different organs. We received five original articles utilizing the next-generation and single-cell sequencing platforms along different organ systems and a review article on CRISPR technology. When utilized with AI, these platforms could help propel new personalized therapies for better patient care. We discuss all the articles in this Research Topic that utilized one or more of these platforms to advance the investigation of disease pathogenesis.

**Obstructive Uropathy:** Bladder outlet obstruction (BOO) is accompanied by fibrosis characterized by smooth muscle hypertrophy and extracellular matrix deposition. Utilizing whole Rat Genome Oligo Microarray on bladder tissue from rats subjected to BOO or control rats, Chen et al. identified the involvement of several inflammatory, dedifferentiation, and fibrosis pathways. Bioinformatic analyses identified survivin which was upregulated in bladder fibrosis during BOO. An increase in survivin expression correlated with the upregulation of cell proliferation and fibrotic protein expression which drove the onset of bladder remodeling. Mechanistically the effects of survivin were through the autophagy pathway as inhibiting autophagy diminished the amelioration of fibrosis and proliferation with pharmacological or genetic inhibition of survivin. Thus, survivin could be a molecular target for an anti-fibrotic therapeutic approach in bladder remodeling during BOO. Combined with organoid technology, this platform can be extended to patients for drug testing and translation into clinical trials.

**Obstructive Pulmonary Disease**: Mastej et al. studied the transcription factors Krüppel-like factor (KLF)-2 and -4 in lung microvasculature. KLF2 was increased whereas KLF4 decreases in lung emphysema. KLF4 and KLF2 form a heterodimer in endothelial cells (ECs). When KLF4 levels fall, this frees KLF2 to bind to its promoter and auto-regulate its expression. EC-specific *Klf4* deficient mice demonstrated enhanced endothelial-to-mesenchymal transition and pulmonary fibrosis. This work has used these disease platforms to highlight the potential role of increasing KLF4 as a therapeutic target in pulmonary fibrosis.

**Genome engineering:** In terms of disease modelling, the last decade has provided huge advances in genetic sequencing; next-generation sequencing (NGS) in particular. The deluge of correlation data of genetic variants with human phenotype has created a ‘bottleneck’ to characterise the functional, and consequently translational, implication of these variants. Llargués-Sistac et al*.* review the potential of gene editing technologies, such as CRISPR-Cas9, and the haploid human cell line HAP1. CRISPR-Cas9 technology utilises guide RNAs, complementary to the target DNA, and a Cas protein nuclease, to edit the host genome. However, technical Research Topic of efficiency and off-target effects remain Research Topic for the field. The HAP1 cell line is a useful partner for high-throughput CRISPR-Cas9 approaches since the haploid nature of the line allows immediate exposure to the effect of any mutation. The authors describe several areas of disease modelling in which the CRISPR-Cas9-HAP1 strategy has led to significant advances, including oncology and immunology.

**Gynecological Complications:** NGS-based technologies have progressed to single-cell analyses, which allows us to display potential unexpected biological discoveries. Ma et al. showcased single-cell transcriptomic datasets of placenta accrete spectrum disorder (PAS), one of the most severe pregnancy complications. They uncovered complex differentiation pathways from progenitor cytotrophoblasts to extravillous trophoblast cells. With the same platform, they revealed complex enhanced crosstalk among various cells and the immune microenvironment during the pathogenesis of the disease. This will aid in deciphering various molecular and cellular pathways, identify novel therapeutic targets, and may pave the way for early prognosis or diagnosis of PAS in clinical practice.

**Ocular Diseases:** AI approaches are improving the efficiency and accuracy of diagnosing many diseases, such as cataracts. A common limitation of AI-based cataract diagnosis models is the poor quality of many fundus images, which can greatly affect the accuracy of the results. Wu et al. now propose a hybrid structure based on a two-stage AI model that could achieve accurate cataract screening even with the interference of poor-quality fundus images. Their anti-interference AI-based-cataract model improved the diagnosis performance by 10% compared with non-anti-interference models.

**Glaucoma** is a progressive disease that affects the optic nerve and leads to irreversible vision loss and blindness. Primary open-angle glaucoma (POAG) is the most common type of glaucoma and is usually asymptomatic in the early stages. Therefore, reliable non-invasive biomarkers are needed for early diagnosis, allowing the implementation of therapeutic strategies that halt the progression of the disease. Network integration analysis uses a broad comprehensive approach that combines multiple sources of biological data to identify the key genes and mechanistic pathways involved in disease pathogenesis. On the other hand, immune infiltration analysis characterizes the different immune cell subtypes hosted in the affected tissues and identifies their potential role in the disease. Using these approaches, Sun et al. aimed at identifying early biomarkers and mechanisms of transcriptome dysregulation in the aqueous humour. They identify significantly differentially expressed genes when comparing healthy subjects and patients with POAG. In addition, three transcription factors (FOS, ATF4, and RELB) were shown to regulate the expression of multiple target genes and T cells regulation. This study sets the foundation for potential therapeutic targets in POAG.

The different research platforms across several organs for disease modelling presented above cover a selection of novel state of art technologies aimed at discovering potential therapeutic targets. In addition, these platforms can be personalized in the era of precision medicine for better patient care.

